# Choline Kinase Alpha2 Promotes Lipid Droplet Lipolysis in Non-Small-Cell Lung Carcinoma

**DOI:** 10.3389/fonc.2022.848483

**Published:** 2022-04-07

**Authors:** Rongxuan Zhu, Yannan Yang, Fei Shao, Juhong Wang, Yibo Gao, Jie He, Zhimin Lu

**Affiliations:** ^1^ Department of Thoracic Surgery, National Cancer Center/National Clinical Research Center for Cancer/Cancer Hospital, Chinese Academy of Medical Sciences and Peking Union Medical College, Beijing, China; ^2^ State Key Laboratory of Molecular Oncology, National Cancer Center/National Clinical Research Center for Cancer/Cancer Hospital, Chinese Academy of Medical Sciences and Peking Union Medical College, Beijing, China; ^3^ Qingdao Cancer Institute, The Affiliated Hospital of Qingdao University, Qingdao University, Qingdao, China; ^4^ Zhejiang Provincial Key Laboratory of Pancreatic Disease, The First Affiliated Hospital, and Institute of Translational Medicine, Zhejiang University School of Medicine, Hangzhou, China; ^5^ Cancer Center, Zhejiang University, Hangzhou, China

**Keywords:** non-small-cell lung cancer, lipid metabolism, choline kinase, phosphorylation, prognostic biomarker

## Abstract

**Background:**

Rapid tumor growth inevitably results in energy stress, including deficiency of glutamine, a critical amino acid for tumor cell proliferation. However, whether glutamine deficiency allows tumor cells to use lipid droplets as an energy resource and the mechanism underlying this potential regulation remain unclear.

**Methods:**

We purified lipid droplets from H322 and H358 human non-small-cell lung cancer (NSCLC) cells under glutamine deprivation conditions and performed immunoblotting to determine the binding of choline kinase (CHK) α2 to lipid droplets. Immunofluorescence was used to quantify lipid droplet numbers and sizes. Immunoprecipitation and immunoblotting were performed to examine AMPK activation and CHKα2 phosphorylation. Cellular fatty acid levels, mitochondrial acetyl coenzyme A and ATP production, and cell apoptosis and proliferation were measured. Immunohistochemical analyses were performed to determine the expression levels of ACC pS79 and CHKα2 pS279 in tumor specimens from NSCLC patients. The prognostic value of ACC pS79 and CHKα2 pS279 was assessed using the Kaplan-Meier method and Cox regression models.

**Results:**

Glutamine deficiency induces AMPK-mediated CHKα2 S279 phosphorylation, which promotes the binding of CHKα2 to lipid droplets, resulting in recruitment of cytosolic lipase ATGL and autophagosomes and subsequent lipolysis of lipid droplets to sustain tumor cell survival and proliferation. In addition, the levels of ACC pS79 and CHKα S279 were much higher in human NSCLC specimens than in their adjacent normal tissues and positively correlated with each other. Notably, ACC pS79 and CHKα pS279 expression levels alone were associated with poor prognosis of NSCLC patients, and combined values of both phosphorylation levels were correlated with worse prognosis of the patients.

**Conclusion:**

CHKα2 plays a critical role in lipolysis of lipid droplets in NSCLC. ACC pS79 and CHKα2 pS279 alone or in combination can be used as prognostic markers in NSCLC.

## Introduction

In recent years, lung cancer-related mortality has shown a significant upward trend worldwide (1), and non-small-cell lung carcinoma (NSCLC) has become the most common pathological type of lung cancer and the leading cause of death among male malignant tumors (2). Rapid growth of tumors, including NSCLC, inevitably induces energy stress. How NSCLC cells reprogram cellular metabolism, especially lipid metabolism, to support tumor cell survival and growth remains largely unclear.

Choline kinase (CHK), which has CHKα (encoded by *CHKA*) and CHKβ (encoded by *CHKB*) isoforms, catalyzes choline to phosphorylcholine. Phosphorylcholine is used for the production of phosphatidylcholine, a main component of the phospholipid bilayer of biological membranes (3). Aberrant upregulation of CHKα expression and elevated levels of its catalytic product phosphorylcholine are detected in a variety of tumors, and CHKα has been shown to play an indispensable role in tumor cell proliferation (4, 5). Of interest, our recent studies demonstrated that CHKα2 possesses noncanonical functions and is involved in hydrolysis of lipid droplets in glioblastoma cells. Under glucose deficiency conditions, AMP-activated protein kinase (AMPK)-mediated CHKα2 phosphorylation at S279 results in KAT5-induced CHKα2 K247 acetylation. Acetylated CHKα2 acts as a protein kinase and phosphorylates PLIN2/3, lipid droplet membrane proteins. PLIN2/3 phosphorylation leads to degradation of these proteins by Hsc70-mediated autophagy, thereby promoting lipid droplet lipolysis and fatty acid oxidation for ATP production and brain tumor cell growth (6). However, whether this regulation also occurs in NSCLC cells under other energy stresses, such as a limited supply of glutamine, which is a critical amino acid for mitochondrial functions and cellular reducing resources (7), remains unclear. In addition, the dynamic regulation between glutamine and lipid droplet metabolism and the importance of this mutual regulation in NSCLC cells have not yet been explored.

In this study, we demonstrated that glutamine deprivation induced AMPK-dependent phosphorylation of CHKα in NSCLC cells. This phosphorylation was indispensable for CHKα to bind to lipid droplets and initiate the lipolysis of lipid droplets for NSCLC cell survival and proliferation.

## Methods

### Specimen Source

Frozen tissues were acquired from 158 pairs of patients with adenocarcinoma/squamous cell carcinoma of the lung who had undergone radical tumor resection in the Department of Thoracic Surgery, Cancer Hospital, Chinese Academy of Medical Sciences, and complete follow-up information was collected. The tissues were collected and extracted with the informed consent of the patients. The project was authorized by the Ethics Committee of the National Cancer Centre/Cancer Hospital, Chinese Academy of Medical Sciences and the Ethics Committee of Peking Union Medical College. **Table 1** summarizes the clinical features of the patients.

**Table 1:** Patient characteristics (N = 316).

Clinicopathological Variables	Number of patients (%)
**Gender**	
Male	189 (59.8%)
Female	127 (40.2%)
**Age, years**	
≤60	143 (45.3%)
>60	173 (54.7%)
**Histology**	
Adenocarcinoma	158 (50.0%)
Squamous cell carcinoma	158 (50.0%)
**Tumor size, cm**	
≤5	137 (43.4%)
>5	179 (56.6%)
**Lymph node metastasis**	
Negative	130 (41.1%)
Positive	186 (58.9%)
**TNM stage**	
Early (I & II)	183 (57.9%)
Late (III & IV)	133 (42.1%)

TNM stage, tumor-node-metastasis stage.

### Cell Culture

HEK 293T, H322 and H358 lung adenocarcinoma cells were obtained from ATCC. HEK 293T and H322 cells grew in DMEM with 10% fetal bovine serum and 1% penicillin streptomycin. H358 cells grew in RPMI 1640 with 10% FBS and 1% penicillin streptomycin. The cells were cultured in 5% CO_2_ at 37°C in a humidified incubator.

### Lentivirus Production and Infection

shRNA targeting the target gene and packaging plasmids were cotransfected into HEK 293T cells using Lipofectamine 3000 transfection reagent. Forty-eight hours later, the virus was harvested and centrifuged. When tumor cells reached 50%-60% confluency, the cells were infected with the concentrated virus and then screened with puromycin treatment. The following shRNA sequences were employed: control shRNA (GCTTCTAACACCGGAGGTCTT), CHKα2 shRNA #1 (TTCTTTCTGAGCTTGTTCG), and CHKα2 shRNA #2 (GTGTTACTTGCAGGTACTTTG).

### IHC

Immunohistochemical (IHC) analysis was performed as described previously (8). The tumor tissue was fixed with paraformaldehyde, embedded in paraffin, and then sliced into sections. The constructed tissue microarray section was stained with antibodies against ACC pS79 and CHKα2 pS279 (9). The immunohistochemical streptavidin-biotin complex (SABC) method was used for the experimental operation. The quantitative scoring of stained tissue sections is presented here with some minor modifications based on previous publications (10). Five nonrepeating fields were randomly selected for each section under high magnification, and Image-Pro Plus 6.0 was used for immunostaining intensity analysis. The yellow–brown area in the immunohistochemical staining image was selected and defined as the area of interest (AOI). The “yellow–brown” color in the image to be measured reflected the amount of corresponding protein expression, and the cumulative absorbance value was calculated by the software to represent protein expression.

### Immunoblotting and Immunoprecipitation Analysis

Proteins were extracted from all NSCLC cell lines treated with and without glutamine deprivation using cell lysis buffer supplemented with cocktail (535142, Sigma, St. Louis, MO, USA). Cell concentrations were quantified using a BCA protein analysis kit (Thermo Fisher Scientific, MA, USA) as described previously (11). Protein electrophoresis was performed with an 8%-12% SDS-PAGE gel according to the molecular weight of the target protein, and then, the proteins in the gel were transferred to a polyvinylidene fluoride (PVDF, Millipore, MA, USA) membrane. Primary antibodies were incubated overnight at 4°C. After incubation with secondary antibody (diluted at 1:5,000) for 1 h, washing was carried out. BeyoECL Plus (Beyotime Biotechnology, Shanghai, China) was used for development and signal detection using an enhanced chemiluminescence system (Pierce, USA).

The cell extract was immunoprecipitated with the specified antibody. After incubation overnight at 4°C, agarose beads were added and incubated for another 3 hours. The immune complexes were washed 3 times with lysis buffer, and then, immunoblot analysis was performed with the corresponding antibodies as described above.

### Lipid Drop Staining

Lipid droplets were isolated as described previously (6). BODIPY was diluted with DMEM (2 μM) and incubated with the cells before and after treatment at 37°C under dark conditions for 15 minutes. After 3 washes, the cells were fixed in 4% paraformaldehyde at room temperature for 1 hour. Then, the cells were photographed under a laser confocal microscope, and the number of lipid droplets and the percentage of the area occupied by lipid droplets were quantitatively analyzed.

### Measurement of ATP Levels

Cellular ATP levels were measured using a colorimetric/fluorimetric kit (BioVision) according to the manufacturer’s instructions as described previously (12).

### Apoptosis Analysis

Apoptosis levels were measured using the TUNEL System (Promega) according to the manufacturer’s instructions as described previously (13).

### Measurement of Cellular Acetyl-CoA Levels

Cell acetyl-CoA levels were measured before and after treatment using the acetyl-CoA fluorometric kit (BioVision) as per the manufacturer’s instructions as described previously (14).

### Determination of Cellular Free Fatty Acids

Cellular free fatty acids were measured using a free fatty acid quantitative colorimetry/fluorescence kit (Biovision) according to the manufacturer’s instructions as described previously (15).

### Statistical Analysis

Data were analyzed using the SPSS 13.0 software package. Data are expressed as the mean ± standard deviation and were compared between two groups by t tests. Differences between multiple groups were analyzed by ANOVA, and correlations were identified by regression analysis. p<0.05 was defined as a significant difference.

## Results

### Glutamine Deprivation Induces the Binding of CHKα2 to Lipid Droplets and Subsequently Promotes CHKα2-Dependent Lipid Droplet Lipolysis in NSCLC Cells

**Figure 1:**
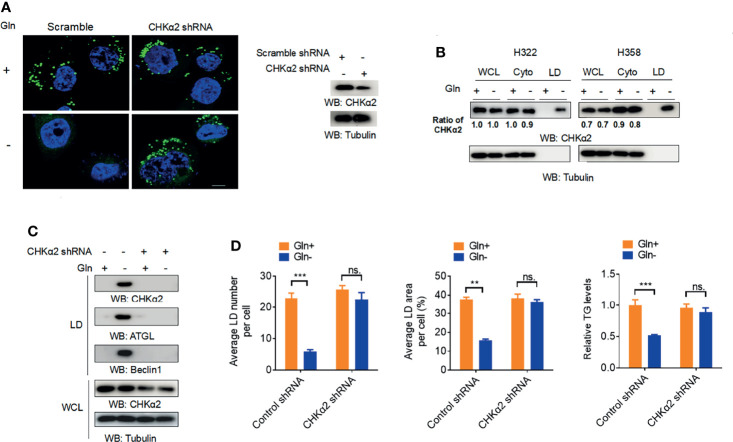
Glutamine deprivation induces the binding of CHKα2 to lipid droplets and subsequently CHKα2-dependent lipid droplet lipolysis in NSCLC cells. **(A)** H322 cells were stimulated with glutamine deprivation with or without expression of CHKα2 shRNA for 6 h. Representative images of lipid droplets are shown (left panel). Immunoblotting with the indicated antibodies was performed (right panel). Scale bar: 10 μm. **(B)** CHKα2 expression in whole cell lysate (WCL), cell cytoplasm (Cyto), and lipid droplets (LD) was examined by stimulating H322 and H258 cells with glutamine deprivation for 6 h. **(C)** H322 cells with or without CHKα2 shRNA expression were stimulated with glutamine deprivation for 6 h. Expression levels of the indicated proteins were detected in WCL or purified lipid droplet samples. **(D)** H322 cells with or without expression of CHKα2 shRNA were stimulated with glutamine deprivation for 6 h. The number and area of lipid droplets were quantified after staining of cells using BODIPY, and relative TG levels were counted. Data represent the mean ± SD of 3 independent experiments. ns, not significant; **P < 0.01; ***P < 0.001.

To examine whether extracellular glutamine levels regulate lipid droplet metabolism in NSCLC cells, we cultured H322 human NSCLC cells in medium deprived of glutamine. Glutamine deprivation substantially reduced the cellular numbers and sizes of lipid droplets (**Figure 1A**). Immunoblotting analyses of purified lipid droplets from H322 and H358 human NSCLC cells revealed that glutamine deprivation induced the binding of a small fraction of CHKα2 in the cytoplasm to lipid droplets (**Figure 1B**). In addition, glutamine deprivation induced the binding of adipose triglyceride lipase (ATGL) and the autophagic protein Beclin1 to lipid droplets (**Figure 1C**) and decreased triglyceride (TG) levels in H322 cells (**Figure 1D**), indicating the hydrolysis of lipid droplets by lipase and autophagy. Notably, depletion of CHKα2, which reduced its binding to lipid droplets, decreased the association of ATGL and Beclin1 with lipid droplets upon glutamine deprivation (**Figure 1C**). Consistent with this finding, CHKα2 depletion blocked the glutamine deprivation-induced decrease in lipid droplets and TG levels (**Figure 1D**). These results indicated that glutamine deprivation induces the binding of CHKα2 to lipid droplets and subsequently promotes CHKα2-dependent lipid droplet lipolysis in NSCLC cells.

### Glutamine Deprivation-Induced and AMPK-Mediated CHKα2 S279 Phosphorylation Promotes the Binding of CHKα2 to Lipid Droplets

**Figure 2:**
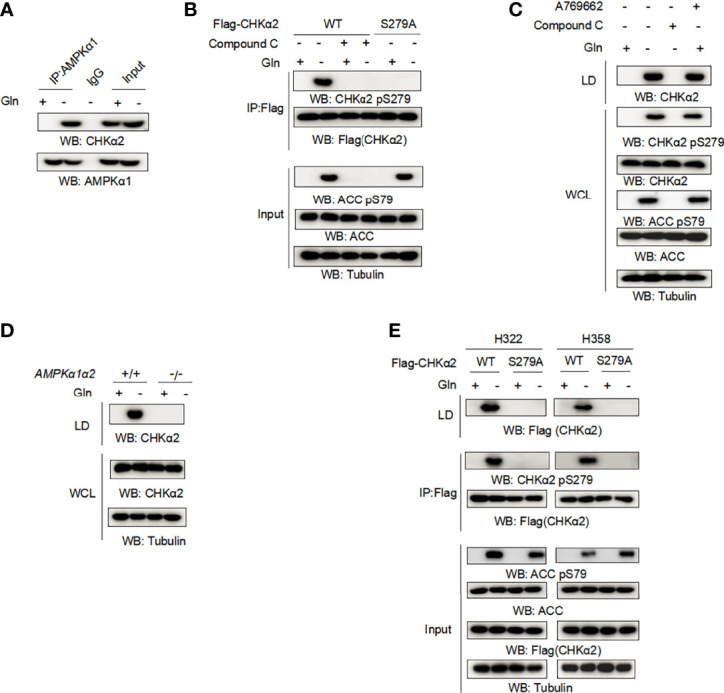
Glutamine deprivation-induced and AMPK-mediated CHKα2 S279 phosphorylation promotes the binding of CHKα2 to lipid droplets. **(A)** H322 cells were stimulated with glutamine deprivation for 6 h. Immunoprecipitation with AMPKα1 antibody and immunoblotting analyses with the indicated antibodies were performed. **(B)** H322 cells expressing WT-Flag-CHKα2 or Flag-CHKα2 S279A were treated with or without 5 μM Compound C for 6 h, followed by stimulation with glutamine deprivation for 6 h. Flag-CHKα2 protein was immunoprecipitated, and immunoblotting analyses with the indicated antibodies were performed. **(C)** H322 cells were treated with 5 μM Compound C or 0.5 mM A769662 for 6 h and stimulated with glutamine deprivation for 6 h. The lipid droplets were purified and normalized to cell numbers. Immunoblotting analyses were performed with the indicated antibodies. **(D)** AMPKα1α2-deficient MEFs were stimulated by glutamine deprivation for 6 h before lipid droplet purification. Immunoblotting analyses were performed with the indicated antibodies. **(E)** The indicated cells expressing WT-Flag-CHKα2 or Flag-CHKα2 pS279A were stimulated with glutamine deprivation for 6 h. Lipid droplets were purified and normalized to cell numbers. Immunoprecipitation of whole cell lysates was performed with an anti-Flag antibody.

Glucose deprivation induced AMP-activated protein kinase (AMPK)-dependent CHKα2 S279 phosphorylation. To determine whether AMPK is involved in the glutamine deficiency-regulated association of CHKα2 with lipid droplets, we performed coimmunoprecipitation assays and showed that glutamine deprivation induced an interaction between endogenous AMPKα1 and endogenous CHKα2 (**Figure 2A**). In addition, glutamine deprivation enhanced CHKα2 S279 phosphorylation, which was abrogated by treatment with the AMPK inhibitor Compound C or expression of the CHKα2 S279A mutant (**Figure 2B**). The inhibition of AMPK by Compound C was reflected by its inhibition of acetyl-CoA carboxylase (ACC) pS79, a known substrate phosphorylation by AMPK. As expected, Compound C treatment blocked the binding of CHKα2 to lipid droplets, whereas treatment with the AMPK activator A769662 induced the association of CHKα2 with lipid droplets even in the absence of glutamine deprivation (**Figure 2C**). Consistent results showed that AMPKα1 and α2 deficiency in mouse embryonic fibroblasts (MEFs) (**Figure 2D**) or CHKα2 S279A mutation (**Figure 2E**) in both H322 and H358 cells abrogated glutamine deprivation-induced recruitment of CHKα2 to lipid droplets. These results indicated that glutamine deprivation-induced and AMPK-mediated CHKα2 S279 phosphorylation promotes the binding of CHKα2 to lipid droplets.

### CHKα2 Promotes Lipid Droplet Lipolysis and Survival of NSCLC Cells

**Figure 3:**
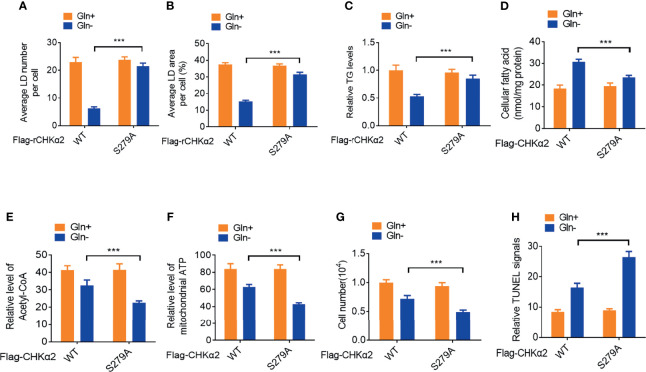
CHKα2 promotes lipid droplet lipolysis and survival of NSCLC cells. **(A–C)** Quantitative analyses of lipid droplet numbers, areas and relative TG levels in the endogenous CHKα2-depleted H322 cells expressing Flag-rCHKα2 WT or S279A were performed. **(D–F)** Cellular fatty acids and levels of mitochondrial acetyl-CoA and ATP were measured in the endogenous CHKα2-depleted H322 cells with Flag-rCHKα2 WT or S279A expression. **(G, H)** Cell numbers were counted, and TUNEL analyses were performed to measure cell death in the endogenous CHKα2-depleted H322 cells with Flag-rCHKα2 WT or S279A expression. ***P < 0.001.

Lipid droplet lipolysis plays a critical role in energy production to sustain cellular activities and the survival of tumor cells (16). Depletion of endogenous CHKα2 and reconstituted expression of RNA interference-resistant (r) wild-type (WT) Flag-rCHKα2 or the Flag-rCHKα2 S279A mutant in H322 cells showed that the CHKα2 S279A mutant strongly inhibited glutamine deprivation-induced decrease of the numbers (**Figure 3A**) and size (**Figure 3B**) of lipid droplets and TG levels (**Figure 3C**). In addition, CHKα2 S279A expression reduced glutamine deprivation-increased cellular fatty acid levels (**Figure 3D**) and aggravated the decrease in acetyl-coenzyme A (**Figure 3E**) and ATP (**Figure 3F**) levels in H322 cells. Correspondingly, CHKα2 S279A expression exacerbated glutamine deprivation-induced cell proliferation (**Figure 3G**) and apoptosis (**Figure 3H**). These results indicated that CHKα2 promotes lipid droplet lipolysis and the survival of NSCLC cells.

### ACC pS79 and CHKα2 pS279 Levels Are Positively Correlated With Poor Prognosis of NSCLC Patients

**Figure 4:**
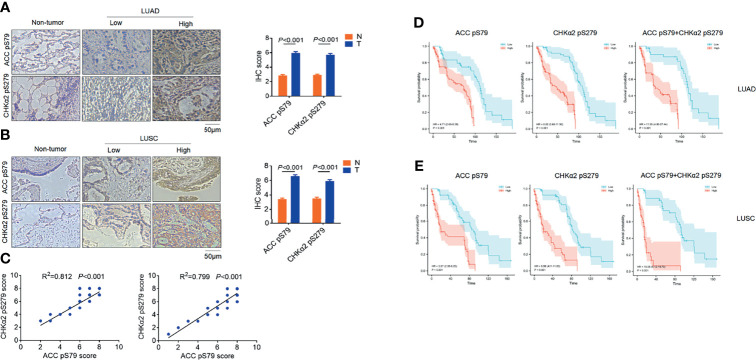
ACC pS79 and CHKα2 pS279 levels are positively correlated with poor prognosis in NSCLC patients. **(A, B)** Representative IHC staining of ACC pS79 and CHKα2 pS279 in LUAD, LUSC, and adjacent non-tumor tissues (left panel). The expression levels of ACC pS79 and CHKα2 pS279 in the NSCLC and adjacent non-tumor tissues were quantified (right panel). **(C)** The correlations between the expression levels of ACC pS79 and CHKα2 pS279 in LUAD (left panel) and LUSC (right panel) specimens were calculated. **(D)** Analysis of the expression levels of ACC pS79, CHKα2 pS279, and combined ACC pS79 and CHKα2 pS279 with the prognosis of LUAD patients was performed. **(E)** Analysis of the expression levels of ACC pS79, CHKα2 pS279, and combined ACC pS79 and CHKα2 pS279 with the prognosis of LUSC patients was performed.

To determine the clinical relevance of AMPK phosphorylation- and CHKα2-mediated lipid droplet lipolysis with NSCLC progression, we performed immunohistochemistry (IHC) analyses of NSCLC specimens (N=316), which included tissue samples of lung adenocarcinoma (LUAD) (N=158, **Figure 4A**) and lung squamous cell carcinoma (N = 158, **Figure 4B**), and adjacent non-neoplastic tissues. We showed that ACC pS79 and CHKα2 pS279 were mainly localized in the cytoplasm of NSCLC tissues. In addition, the expression levels of ACC pS79 and CHKα2 pS279 were much higher in the NSCLC tissues than in the adjacent non-neoplastic tissues (**Figures 4A, B**). In addition, the expression levels of ACC pS79 and CHKα2 pS279 in LUAD and LUSC specimens were positively correlated with each other (**Figure 4C**).

We next examined the relationship between the expression levels of ACC pS79 and CHKα2 pS279 and the clinical aggressiveness of NSCLC and showed that the levels of ACC pS79 and CHKα2 pS279 were not statistically correlated with patient sex, age, or tumor size (P > 0.05, **Table 2**). Notably, both ACC pS79 and CHKα2 pS279 expression levels were associated with pathological type, lymph node metastasis and advanced TNM stage (P < 0.05, **Table 2**). In addition, high levels of ACC pS79 and CHKα2 pS279 were positively correlated with poor prognosis of patients with LUAD (**Figure 4D**) and LUSC (**Figure 4E**). In line with this finding, the combined expression levels of ACC pS79 and CHKα2 pS279 were also inversely correlated with the survival time of the patients with LUAD (**Figure 4D**) and LUSC (**Figure 4E**).

**Table 2:** Correlation analyses of ACC pS79 and CHKα2 pS279 expressions with clinic-pathological characteristics.

Clinicopathological Variables	N	ACC pS79 Expression		*P V*alue	CHKα2 pS279 Expression		P Value
		Low (127)	High (189)		Low (134)	High (182)	
**Gender**							
Male	189	72	117	0.354	80	109	0.974
Female	127	55	72		54	73
**Age, years**							
≤60	143	58	85	0.903	55	88	0.197
>60	173	69	104		79	94
**Histology**							
Adenocarcinoma	158	77	81	0.002	81	77	<0.001
Squamous cell carcinoma	158	50	108		53	105
**Tumor size, cm**							
≤5	137	61	76	0.169	51	86	0.103
>5	179	66	113		83	96
**Lymph node metastasis**							
Negative	130	64	66	0.019	42	88	0.002
Positive	186	67	119		92	94
**TNM stage**							
Early (I & II)	183	92	91	<0.001	90	93	0.004
Late (III & IV)	133	35	98		44	89

Univariate Cox regression analyses revealed that high expression levels of ACC pS79, CHKα2 pS279, and combined ACC pS79 and CHKα2 pS279 were associated with a shorter overall survival time in patients with LUAD and LUSC (**Table 3**). Notably, the combined expression values of ACC pS79 and CHKα2 pS279 in LUAD (hazard ratio [HR]: 11.55; 95% confidence interval [CI], 4.86-27.44) were a better prognostic predictor than ACC pS79 (HR: 4.71; 95% CI, 2.65-8.39) or CHKα2 pS279 (HR: 6.62; 95% CI, 3.68-11.90) alone. Similarly, the combined expression values of ACC pS79 and CHKα2 pS279 in LUSC (HR; 10.05; 95% CI, 5.12-19.73) were also a better prognostic predictor than ACC pS79 expression (HR; 3.87; 95% CI, 2.39-6.25) or CHKα2 pS279 expression (HR: 6.98; 95% CI, 4.11-11.83) alone.

**Table 3:** Univariate and multivariate Cox regression analyses of risk factors associated with overall survival.

Variables	Univariate analysis			Multivariate analysis		
	HR	95% CI	*P* Value	HR	95% CI	*P* Value
Characteristics (LUAD)						
ACC pS79 Expression (High vs. Low)	4.71	2.65-8.39	<0.001	4.53	2.56-8.04	<0.001
CHKα2 pS279 Expression (High vs. Low)	6.62	3.68-11.90	<0.001	6.39	3.57-11.41	<0.001
ACC pS79 +CHKα2 pS279 Expression	11.55	4.86-27.44	<0.001	10.97	4.67-25.74	<0.001
Gender (female vs. male)	1.09	0.72-1.64	0.69	\	\	\
Age (>60 vs. ≤60)	0.73	0.47-1.11	0.14	\	\	\
TNM stage (Late vs. Early)	2.76	1.78-2.49	<0.001	\	\	\
Characteristics (LUSC)						
ACC pS79 Expression (High vs. Low)	3.87	2.39-6.25	<0.001	3.76	2.33-6.08	<0.001
CHKα2pS279 Expression (High vs. Low)	6.98	4.11-11.83	<0.001	6.87	4.05-11.67	<0.001
ACC pS79 +CHKα2 pS279 Expression	10.05	5.12-19.73	<0.001	10.46	5.62-23.33	<0.001
Gender (female vs. male)	0.98	0.65-1.49	0.69	\	\	\
Age (>60 vs. ≤60)	0.92	0.61-1.40	0.70	\	\	\
TNM stage (Late vs. Early)	1.81	1.21-2.83	<0.001	\	\	\

Consistent with the results from univariate Cox regression analyses, the multivariate analysis of patients with NSCLC showed that the levels of ACC pS79 or CHKα2 pS279 (HR: 4.531; 95% CI, 2.562-8.04 for ACC pS79 in LUAD; HR: 3.766; 95% CI, 2.332-6.081 for ACC pS79 in LUSC; HR, 6.392; 95% CI, 3.579-11.415 for CHKα2 pS279 in LUAD; HR: 6.878; 95% CI, 4.053-11.670 for CHKα2 pS279 in LUSC) were also independent prognostic markers for NSCLC. In addition, the combined expression values of ACC pS79 and CHKα2 pS279 exhibited a better prognostic value than each phosphorylation alone (HR: 10.966; 95% CI, 4.671-25.744 in LUAD and HR: 11.460; 95% CI, 5.627-23.339 in LUSC). These results indicated that the levels of ACC pS79, CHKα2 pS279, or combined ACC pS79 and CHKα2 pS279 are independent prognostic factors for patients with NSCLC (**Table 3**).

## Discussion

Tumor cells, including NSCLC cells, not only actively synthesize lipids for cell growth and proliferation but also initiate lipid droplet lipolysis to counteract energy stress with less characterized mechanisms (17–19). We revealed that glutamine deficiency induces AMPK-dependent CHKα2 S279 phosphorylation. This phosphorylation promotes the binding of CHKα2 to lipid droplets. CHKα2 S279 phosphorylation resulted in recruitment of cytosolic lipase ATGL and autophagosomes and subsequent lipolysis of lipid droplets to sustain tumor cell survival and proliferation. In addition, the levels of ACC pS79 and CHKα2 S279 were much higher in the human NSCLC specimens than in their adjacent normal tissues and positively correlated with each other. Importantly, ACC pS79 and CHKα2 pS279 expression levels alone were associated with poor prognosis of NSCLC patients, and combined values of both phosphorylation levels exhibited a better prognostic value.

Metabolic enzymes can possess noncanonical functions, which are critical for tumor progression (20–26). CHKα2 is a metabolic enzyme originally defined as a phosphocholine kinase for PC production and is overexpressed in tumor cells. Our recent studies revealed that CHKα2 can act as a protein kinase to phosphorylate PLIN2/3 and initiate lipid droplet lipolysis for tumor growth. Given that disruption of canonical CHKα2 functions inevitably affects choline phosphorylation and normal cell proliferation, targeting the moonlighting functions of CHKα2 to promote lipolysis can be exploited to mitigate tumor progression. Our findings that CHKα2 plays a critical role in lipolysis of lipid droplets in NSCLC suggest an attractive strategy to target the protein kinase activity of CHKα2 for NSCLC treatment.

## Data Availability Statement

The original contributions presented in the study are included in the article/supplementary material. Further inquiries can be directed to the corresponding authors.

## Ethics Statement

This study was approved by the Institute Research Medical Ethics Committee of the National Cancer Center/National Clinical Research Center for Cancer/Cancer Hospital, Chinese Academy of Medical Sciences and Peking Union Medical College in Beijing. Paired carcinoma and normal tissue specimens were obtained from the National Cancer Center/National Clinical Research Center for Cancer/Cancer Hospital, Chinese Academy of Medical Sciences and Peking Union Medical College in Beijing. All tissue samples were collected in compliance with an informed consent policy. Written informed consent was obtained from all the patients at the time of admission for the use of their tissues for scientific research, and patient privacy was protected. The patients/participants provided their written informed consent to participate in this study.

## Author Contributions

ZL conceived and designed the study. JH provided critical scientific input. RZ, YY, FS, JW, and YG performed the experiments. RZ wrote the draft manuscript. ZL and RZ revised the manuscript. All authors contributed to the article and approved the submitted version.

## Funding

This study was supported by grants from the Ministry of Science and Technology of the People’s Republic of China (2020YFA0803300, ZL), the National Natural Science Foundation of China (82188102, 82030074, ZL; 82122053, YG; 32100574, FS), R&D Program of Beijing Municipal Education commission (KJZD20191002302, JH), CAMS Initiative for Innovative Medicine (2021-1-I2M-012, JH), Aiyou Foundation (KY201701, JH), the Natural Science Foundation of Shandong Province (ZR2020QH191, FS), Zhejiang Natural Science Foundation-Key Project (LD21H160003, ZL), and the Leading Innovative and Entrepreneur Team Introduction Program of Zhejiang (2019R01001, ZL). ZL is the Kuancheng Wang Distinguished Chair.

## Conflict of Interest

ZL owns shares in Signalway Biotechnology (Pearland, TX), which supplied rabbit antibodies that recognize CHKα2 pS279. ZL’s interest in this company had no bearing on its being chosen to supply these reagents.

The remaining authors declare that the research was conducted in the absence of any commercial or financial relationships that could be construed as a potential conflict of interest.

## Publisher’s Note

All claims expressed in this article are solely those of the authors and do not necessarily represent those of their affiliated organizations, or those of the publisher, the editors and the reviewers. Any product that may be evaluated in this article, or claim that may be made by its manufacturer, is not guaranteed or endorsed by the publisher.
